# HLA-B*46:01:01:01 and HLA-DRB1*09:01:02:01 are associated with anti-rHuEPO-induced pure red cell aplasia

**DOI:** 10.1038/s41598-023-50211-3

**Published:** 2023-12-20

**Authors:** Thitima Benjachat Suttichet, Monpat Chamnanphon, Monnat Pongpanich, Sarun Chokyakorn, Pawinee Kupatawintu, Chalurmpon Srichomthong, Wanna Chetruengchai, Hathaichanok Chuntakaruk, Thanyada Rungrotmongkol, Pajaree Chariyavilaskul, Vorasuk Shotelersuk, Kearkiat Praditpornsilpa

**Affiliations:** 1 Center of Excellence in Clinical Pharmacokinetics and Pharmacogenomics, Faculty of Medicine, Chulalongkorn University, Bangkok, Thailand; 2 Department of Mathematics and Computer Science, Faculty of Science, Chulalongkorn University, Bangkok, Thailand; 3 Faculty of Science, Omics Sciences and Bioinformatics Center, Chulalongkorn University, Bangkok, Thailand; 4 Department of Pharmacology, Faculty of Medicine, Chulalongkorn University, Bangkok, Thailand; 5 National Blood Center, Thai Red Cross Society, Bangkok, Thailand; 6 Department of Pediatrics, Faculty of Medicine, Center of Excellence for Medical Genomics, Chulalongkorn University, Bangkok, Thailand; 7 Excellence Center for Genomics and Precision Medicine, King Chulalongkorn Memorial Hospital, The Thai Red Cross Society, Bangkok, Thailand; 8 Program in Bioinformatics and Computational Biology, Graduate School, Chulalongkorn University, Bangkok, Thailand; 9 Department of Biochemistry, Faculty of Science, Center of Excellence in Structural and Computational Biology, Chulalongkorn University, Bangkok, Thailand; 10 Division of Nephrology, Department of Medicine, Faculty of Medicine, Chulalongkorn University, Bangkok, Thailand

**Keywords:** Medical research, Genetic predisposition to disease, Nephrology, Kidney diseases

## Abstract

Treatment of anemia in patients with chronic kidney disease (CKD) with recombinant human erythropoietin (rHuEPO) can be disrupted by a severe complication, anti-rHuEPO-induced pure red cell aplasia (PRCA). Specific HLA genotypes may have played a role in the high incidence of PRCA in Thai patients (1.7/1,000 patient years vs. 0.03/10,000 patient years in Caucasians). We conducted a case–control study in 157 CKD patients with anti-rHuEPO-induced PRCA and 56 controls. The HLA typing was determined by sequencing using a highly accurate multiplex single-molecule, real-time, long-read sequencing platform. Four analytical models were deployed: Model 1 (additive: accounts for the number of alleles), Model 2 (dominant: accounts for only the presence or absence of alleles), Model 3 (adjusted additive with rHuEPO types) and Model 4 (adjusted dominant with rHuEPO types). HLA-B*46:01:01:01 and DRB1*09:01:02:01 were found to be independent risk markers for anti-rHuEPO-induced PRCA in all models [OR (95%CI), p-values for B*46:01:01:01: 4.58 (1.55–13.51), 0.006; 4.63 (1.56–13.75), 0.006; 5.72 (1.67–19.67), 0.006; and 5.81 (1.68–20.09), 0.005; for DRB1*09:01:02:01: 3.99 (1.28–12.49), 0.017, 4.50 (1.32–15.40), 0.016, 3.42 (1.09–10.74), 0.035, and 3.75 (1.08–13.07), 0.038, in Models 1–4, respectively. HLA-B*46:01:01:01 and DRB1*09:01:02:01 are susceptible alleles for anti-rHuEPO-induced PRCA. These findings support the role of HLA genotyping in helping to monitor patients receiving rHuEPO treatment.

## Introduction

Patients with advanced chronic kidney disease (CKD) develop anemia, in part due to insufficient endogenous erythropoietin (EPO) production related to the progression of CKD^1^. Recombinant human erythropoietin (rHuEPO) was an erythropoiesis stimulating agent (ESA) introduced into clinical practice to treat anemia related to CKD in the 1990s and successfully improved clinical outcomes and quality of life of patients with CKD^1^. However, reports have shown that rHuEPO was associated with a severe complication known as anti-rHuEPO-induced pure red cell aplasia (PRCA), a rapid-onset normocytic-normochromic anemia, and severe reticulocytopenia resistant to rHuEPO^1–13^.

PRCA is caused by EPO-induced antibodies that neutralize all exogenous drugs and cross-react with endogenous EPO, leading to undetectable serum levels of EPO, ineffective erythropoiesis, and blood transfusion-dependent to relieve anemic symptoms^8,11,14,15^. Factors related to an increase in the incidence of PRCA included stabilizer, immunogenic polysorbate 80 that was used in human serum albumin-forming micelles, and leachates released by an uncoated rubber prefilled syringe stopper that can interact with polysorbate 80 that aggravating immune reactions^8,15–17^. Other factors were problems related to EPO product quality, the cold chain, and the subcutaneous route of administration^5,6,8,17^. Furthermore, immunogenicity is partly dependent on the type of genetic variations in human leukocyte antigen (HLA)^17^. Previous studies have shown that the mechanism of PRCA may be related to antibody response in genetically susceptible patients (HLA-DRB1*09)^2–5,7,9^.

The Thai ESA registry reported that the incidence of anti-rHuEPO-induced PRCA in Thai patients was at least 1.7 per 1000 patient years^18^. This incidence is much higher than the incidence of anti-rHuEPO-induced PRCA in western countries^2,4,9–12,18–20^. Our previous data^4^ and other groups^3,7^ suggested the role of HLA-DRB1*09-DQB1*03:09 and HLA DRB1*12:02 as risk factors for anti-rHuEPO-induced PRCA. However, studies^3,4,7^ were conducted in a small group of cases (24, 22, and 8 cases, respectively) using an intermediate resolution HLA genotyping technique; therefore, the exact relationships of all the genetic variability of HLA with anti-rHuEPO-induced PRCA cannot be fully determined.

In addition to the factors mentioned above, numerous rHuEPO biocopy products are widely used in Thailand and other developing countries. Our previous report showed that rHuEPO biocopy products were associated with neutralizing antibody production and PRCA^19^. The rHuEPO biocopy products differ in a substantial amount of their protein fragments^21^, which can also play a crucial role in anti-rHuEPO-induced PRCA. However, this factor has not yet been thoroughly clinically evaluated.

This study investigated associations of HLA genotypes, sequenced by highly accurate long-read HLA sequencing, with anti-rHuEPO-induced PRCA in Thai patients with CKD. Relationships were also adjusted for the source of rHuEPO products (innovator and biocopy).

## Results

### Baseline characteristics and frequency of HLA alleles in cases and controls

A total of 213 participants (157 cases and 56 controls) were enrolled. As expected, the cases had a significantly shorter exposure time to rHuEPO compared to the controls. All participants had Telfon-coated prefilled rHuEPO administered subcutaneously. Two brands of innovator rHuEPO were prescribed to 14 cases (8.92%), while none of the controls received innovator rHuEPO. At least nine brands of rHuEPO biocopy were prescribed in both cases and controls (Table 1).

**Table 1:** Baseline characteristics.

Parameters	Cases (n = 157)	Controls (n = 56)	p-value
Age [years, mean (range)]	64 (21–87)	42 (22–72)	0.002
Male/female (n (%))	112/45 (71/29)	31/25 (55/45)	n/a
Exposure time [months; mean (range)]	9.3 (0.1–40.1)	69.9 (31.1–281.4)	0.000
Subcutaneous Teflon-coated prefilled rHuEPO [n (%)]	100 (100.00)	100 (100.00)	n/a
Types of rHuEPO* [n (%)]			
Innovator	14 (8.92)	0 (0.00)	n/a
Innovator 1	7 (4.46)	0 (0.00)	n/a
Innovator 2	7 (4.46)	0 (0.00)	n/a
Biosimilar	0 (0.00)	0 (0.00)	n/a
Biocopy	143 (91.08)	56 (100.00)	n/a
Biocopy 1	1 (0.64)	0 (0.00)	n/a
Biocopy 2	1 (0.64)	3 (5.36)	n/a
Biocopy 3	1 (0.64)	0 (0.00)	n/a
Biocopy 4	1 (0.64)	0 (0.00)	n/a
Biocopy 5	11 (7.01)	7 (12.50)	n/a
Biocopy 6	29 (18.47)	1 (1.79)	n/a
Biocopy 7	21 (13.38)	32 (57.14)	n/a
Biocopy 8	50 (31.85)	2 (3.57)	n/a
Biocopy 9	6 (3.82)	0 (0.00)	n/a
Unknown	22 (14.01)	11 (19.64)	n/a

*rHuEPO* recombinant human erythropoietin.

*The rHuEPO innovator products were defined as the first drugs created containing its specific active ingredient to receive approval for use^31,32^. The rHuEPO biosimilar products were defined according to the guidelines of the United States Food & Drug Administration^31^ and/or the European Medicines Agency^32^. Generic rHuEPO products that did not meet these criteria were considered rHuEPO biocopy products.

Tables 2, 3 and 4 summarize the frequency of the HLA alleles observed in the population studied. For HLA Class I, 34, 68, and 32 HLA alleles were identified for HLA-A, HLA-B, and HLA-C, respectively (Table 2). The HLA Class I alleles highly present in cases and controls were HLA-A*11:01:01:01 (19.20% and 24.26%), HLA-B*46:01:01:01 (16.07% and 7.14%), and HLA-C*01:02:01:01 (17.82% and 13.47%), (Table 2). Furthermore, 37, 10, 5, 28, and 43 HLA Class II alleles were observed for HLA-DRB1, HLA-DRB3, HLA-DRB4, HLA-DQB1, and HLA-DPB1, respectively (Tables 3 and 4). HLA Class II alleles that were highly observed in cases and controls were HLA-DRB1*12:02:01:01 (16.94% and 18.24%), HLA-DRB3*02:02:01:01 (35.59% and 34.38%), HLA-DRB4*01:03:01:05 (43.55% and 47.22%), HLA-DQB1*05:02:01:01 (24.91% and 19.76%) and HLA-DPB1*05:01:01:01 (19.46% and 28.15%), (Tables 3 and 4).

**Table 2:** Frequency of HLA-A, HLA-B, and HLA-C alleles in cases and controls.

HLA-A	Cases		Controls		HLA-B	Cases		Controls		HLA-B	Cases		Controls		HLA-C	Cases		Controls	
	n	%	n	%		n	%	n	%		n	%	n	%		n	%	n	%
A*01:01:01:01	6	2.17	4	2.94	B*07:02:01:01	4	1.43	2	1.43	B*39:02:02:04	0	0.00	2	1.43	C*01:02:01:01	49	17.82	18	13.74
A*02:01:01:01	3	1.09	10	7.35	B*07:05:01:01	0	0.00	1	0.71	B*39:09:01:01	8	2.86	1	0.71	C*02:02:02:01	1	0.36	0	0.00
A*02:03:01:01	30	10.87	12	8.82	B*07:06:01:01	13	4.64	3	2.14	B*39:188	0	0.00	1	0.71	C*03:02:02:01	21	7.64	9	6.87
A*02:06:01:01	7	2.54	1	0.74	B*08:01:01:02	1	0.36	2	1.43	B*39:77	0	0.00	1	0.71	C*03:03:01:01	5	1.82	1	0.76
A*02:07:01:01	34	12.32	14	10.29	B*13:01:01:01	18	6.43	5	3.57	B*40:01:02:01	17	6.07	9	6.43	C*03:04:01:02	29	10.55	7	5.34
A*02:10	0	0.00	1	0.74	B*13:01:01:07	1	0.36	0	0.00	B*40:02:01:01	5	1.79	1	0.71	C*03:04:01:18	1	0.36	1	0.76
A*02:11:01:01	2	0.72	2	1.47	B*13:02:01:01	3	1.07	0	0.00	B*40:02:01:21	1	0.36	0	0.00	C*03:04:04	0	0.00	1	0.76
A*02:131:01:01	1	0.36	0	0.00	B*15:01:01:01	4	1.43	0	0.00	B*40:03:01:02	1	0.36	0	0.00	C*03:17:01	1	0.36	0	0.00
A*02:20:02	1	0.36	0	0.00	B*15:02:01:01	16	5.71	14	10.00	B*40:06:01:01	3	1.07	1	0.71	C*04:01:01:01	10	3.64	6	4.58
A*03:01:01:01	2	0.72	1	0.74	B*15:06	2	0.71	0	0.00	B*40:06:01:02	1	0.36	1	0.71	C*04:01:01:06	0	0.00	1	0.76
A*03:02:01:01	0	0.00	1	0.74	B*15:08:01:01	1	0.36	0	0.00	B*40:06:04:01	0	0.00	2	1.43	C*04:03:01:01	9	3.27	3	2.29
A*11:01:01:01	53	19.20	33	24.26	B*15:109	0	0.00	1	0.71	B*40:12:01	0	0.00	2	1.43	C*04:06:01	7	2.55	2	1.53
A*11:01:01:07	10	3.62	4	2.94	B*15:11:01	1	0.36	0	0.00	B*41:01:01:01	0	0.00	1	0.71	C*04:10	1	0.36	0	0.00
A*11:01:01:22	2	0.72	0	0.00	B*15:12:01	2	0.71	0	0.00	B*44:03:02:01	8	2.86	6	4.29	C*05:01:01:12	0	0.00	1	0.76
A*11:02:01:01	6	2.17	5	3.68	B*15:13:01	0	0.00	3	2.14	B*44:06	0	0.00	1	0.71	C*06:02:01:01	11	4.00	2	1.53
A*24:02:01:01	40	14.49	15	11.03	B*15:18:01:02	0	0.00	1	0.71	B*46:01:01:01	45	16.07	10	7.14	C*07:01:01:01	2	0.73	0	0.00
A*24:02:01:02L	0	0.00	1	0.74	B*15:25:01:01	6	2.14	4	2.86	B*46:88	1	0.36	0	0.00	C*07:02:01:01	41	14.91	22	16.79
A*24:02:40:01	2	0.72	1	0.74	B*15:32:01	6	2.14	1	0.71	B*47:01:01:03	1	0.36	0	0.00	C*07:02:01:03	2	0.73	1	0.76
A*24:03:01:01	4	1.45	0	0.00	B*15:35:01:01	3	1.07	1	0.71	B*48:01:01:01	4	1.43	2	1.43	C*07:02:01:51	1	0.36	1	0.76
A*24:07:01:01	8	2.90	8	5.88	B*15:450	3	1.07	0	0.00	B*48:03:01:01	0	0.00	1	0.71	C*07:04:01:01	15	5.45	6	4.58
A*24:10:01:01	6	2.17	3	2.21	B*18:01:01:02	10	3.57	4	2.86	B*51:01:01:01	11	3.93	5	3.57	C*07:06:01:01	9	3.27	7	5.34
A*24:20:01:01	3	1.09	0	0.00	B*18:02:01	7	2.50	1	0.71	B*51:01:02:01	5	1.79	1	0.71	C*08:01:01:01	21	7.64	19	14.50
A*26:01:01:01	5	1.81	0	0.00	B*27:04:01	2	0.71	4	2.86	B*51:02:01:01	0	0.00	6	4.29	C*08:03:01:01	1	0.36	0	0.00
A*29:01:01:01	0	0.00	1	0.74	B*27:05:02:01	1	0.36	0	0.00	B*52:01:01:01	3	1.07	4	2.86	C*12:02:02:01	9	3.27	7	5.34
A*30:01:01:01	5	1.81	1	0.74	B*27:06:01:01	9	3.21	2	1.43	B*52:01:01:02	1	0.36	1	0.71	C*12:03:01:01	8	2.91	2	1.53
A*31:01:02:01	3	1.09	2	1.47	B*27:07:01	1	0.36	0	0.00	B*54:01:01:01	1	0.36	2	1.43	C*12:301	1	0.36	0	0.00
A*32:01:01:01	0	0.00	1	0.74	B*35:01:01:02	1	0.36	2	1.43	B*55:02:01:02	0	0.00	2	1.43	C*14:02:01:01	11	4.00	5	3.82
A*33:03:01:01	35	12.68	12	8.82	B*35:02:01:01	0	0.00	1	0.71	B*55:02:01:03	5	1.79	0	0.00	C*15:02:01:01	6	2.18	7	5.34
A*34:01:01:01	2	0.72	0	0.00	B*35:03:01:01	0	0.00	1	0.71	B*56:01:01:03	4	1.43	1	0.71	C*15:02:01:58	0	0.00	1	0.76
A*68:01:02:01	0	0.00	1	0.74	B*35:05:01:01	5	1.79	2	1.43	B*56:04:01:01	3	1.07	0	0.00	C*15:04:01:01	1	0.36	0	0.00
A*68:01:02:02	0	0.00	1	0.74	B*35:30:01:01	0	0.00	1	0.71	B*56:10	1	0.36	0	0.00	C*15:05:02:01	0	0.00	1	0.76
A*68:01:02:10	2	0.72	0	0.00	B*37:01:01:01	0	0.00	1	0.71	B*57:01:01:01	5	1.79	1	0.71	C*16:02:01:01	2	0.73	0	0.00
A*74:02:01:02	2	0.72	1	0.74	B*38:02:01:01	3	1.07	8	5.71	B*58:01:01:01	20	7.14	8	5.71	Alleles analyzed	275	100.00	131	100.00
A*74:05	2	0.72	0	0.00	B*39:01:01:03	3	1.07	0	0.00	B*58:01:01:10	0	0.00	2	1.43					
Alleles analyzed	276	100.00	136	100.00						Alleles analyzed	280	100.00	140	100.00

**Table 3:** Frequency of HLA-DRB1, HLA-DRB3, and HLA-DRB4 alleles in cases and controls.

HLA-DRB1	Cases		Controls		HLA-DRB3	Cases		Controls		HLA-DRB4	Cases		Controls	
	n	%	n	%		n	%	n	%		n	%	n	%
DRB1*01:01:01:01	0	0.00	4	2.37	DRB3*01:01:02:01	7	5.93	3	4.69	DRB4*01:03:01:01	17	27.42	16	44.44
DRB1*03:01:01:01	20	6.51	15	8.88	DRB3*01:01:02:04	1	0.85	1	1.56	DRB4*01:03:01:02N	5	8.06	1	2.78
DRB1*04:03:01:01	0	0.00	1	0.59	DRB3*01:14	1	0.85	0	0.00	DRB4*01:03:01:05	27	43.55	17	47.22
DRB1*04:03:02	6	1.95	0	0.00	DRB3*02:02:01:01	42	35.59	22	34.38	DRB4*01:03:02:01	12	19.35	2	5.56
DRB1*04:05:01:01	2	0.65	6	3.55	DRB3*02:02:01:02	13	11.02	7	10.94	DRB4*01:03:03	1	1.61	0	0.00
DRB1*04:05:22	1	0.33	0	0.00	DRB3*02:02:01:06	8	6.78	6	9.38	Alleles analyzed	62	100.00	36	100.00
DRB1*04:06:01	0	0.00	1	0.59	DRB3*03:01:01:01	1	0.85	2	3.13					
DRB1*07:01:01:01	22	7.17	16	9.47	DRB3*03:01:03:01	21	17.80	6	9.38					
DRB1*08:02:01:01	1	0.33	0	0.00	DRB3*03:01:03:02	24	20.34	16	25.00					
DRB1*08:03:02:01	7	2.28	7	4.14	DRB3*03:03	0	0.00	1	1.56					
DRB1*08:09:01	1	0.33	0	0.00	Alleles analyzed	118	100.00	64	100.00					
DRB1*08:12	2	0.65	0	0.00										
DRB1*08:19	1	0.33	2	1.18										
DRB1*09:01:02:01	39	12.70	10	5.92										
DRB1*10:01:01:01	1	0.33	7	4.14										
DRB1*11:01:01:01	5	1.63	6	3.55										
DRB1*11:04:01:01	0	1.95	1	0.00										
DRB1*11:129	6	0.00	0	0.59										
DRB1*12:01:01:01	3	0.98	3	1.78										
DRB1*12:02:01:01	52	16.94	31	18.34										
DRB1*13:01:01:01	2	0.65	1	0.59										
DRB1*13:02:01:01	1	0.33	2	1.18										
DRB1*13:12:01	2	0.65	0	0.00										
DRB1*14:04:01:01	13	4.23	5	2.96										
DRB1*14:05:01:01	3	0.98	3	1.78										
DRB1*14:22	1	0.33	0	0.00										
DRB1*14:54:01:01	19	6.19	6	3.55										
DRB1*14:54:11	1	0.33	0	0.00										
DRB1*15:01:01:01	26	8.47	12	7.10										
DRB1*15:01:05	0	0.00	1	0.59										
DRB1*15:02:01:02	43	14.01	21	12.43										
DRB1*15:02:01:04	5	1.63	2	1.18										
DRB1*15:02:02:01	2	0.65	1	0.59										
DRB1*15:04	1	0.33	0	0.00										
DRB1*15:48	2	0.65	0	0.00										
DRB1*16:02:01:01	13	4.23	4	2.37										
DRB1*16:02:01:06	4	1.30	1	0.59										
Alleles analyzed	307	100.00	169	100.00

**Table 4:** Frequency of HLA-DQB1 and HLA-DPB1 alleles in cases and controls.

HLA-DQB1	Cases		Controls		HLA-DPB1	Cases		Controls	
	n	%	n	%		n	%	n	%
DQB1*02:01:01:01	7	2.42	6	3.59	DPB1*01:01:01:04	4	1.56	2	1.48
DQB1*02:02:01:01	6	2.08	2	1.20	DPB1*02:01:02:01	15	5.84	4	2.96
DQB1*03:01:01:01	4	1.38	1	0.60	DPB1*02:01:02:03	2	0.78	1	0.74
DQB1*03:01:01:02	7	2.42	4	2.40	DPB1*02:01:02:11	2	0.78	1	0.74
DQB1*03:01:01:04	0	0.00	1	0.60	DPB1*02:01:02:32	0	0.00	2	1.48
DQB1*03:01:01:07	24	8.30	19	11.38	DPB1*02:01:02:52	1	0.39	0	0.00
DQB1*03:01:01:12	2	0.69	3	1.80	DPB1*02:01:02:73	0	0.00	1	0.74
DQB1*03:01:01:56	1	0.35	0	0.00	DPB1*02:02:01:01	20	7.78	11	8.15
DQB1*03:02:01:01	10	3.46	10	5.99	DPB1*03:01:01:01	12	4.67	6	4.44
DQB1*03:03:02:01	4	1.38	1	0.60	DPB1*03:01:01:20	1	0.39	0	0.00
DQB1*03:03:02:02	27	9.34	8	4.79	DPB1*03:01:15:01	1	0.39	0	0.00
DQB1*04:01:01:03	10	3.46	12	7.19	DPB1*04:01:01:01	28	10.89	11	8.15
DQB1*04:02:01:04	1	0.35	0	0.00	DPB1*04:01:01:113	1	0.39	0	0.00
DQB1*04:02:01:09	1	0.35	3	1.80	DPB1*04:01:01:41	1	0.39	0	0.00
DQB1*04:51	1	0.35	0	0.00	DPB1*04:01:01:75	1	0.39	0	0.00
DQB1*05:01:01:02	0	0.00	7	4.19	DPB1*04:02:01:02	4	1.56	7	5.19
DQB1*05:01:01:05	1	0.35	6	3.59	DPB1*05:01:01:01	50	19.46	38	28.15
DQB1*05:01:24:01	43	14.88	19	11.38	DPB1*05:01:01:04	4	1.56	0	0.00
DQB1*05:02:01:01	72	24.91	33	19.76	DPB1*09:01:01:01	4	1.56	3	2.22
DQB1*05:03:01:01	16	5.54	7	4.19	DPB1*10:01:01:01	1	0.39	0	0.00
DQB1*05:03:01:04	1	0.35	0	0.00	DPB1*104:01:01:01	0	0.00	1	0.74
DQB1*05:165	1	0.35	0	0.00	DPB1*104:01:01:03	1	0.39	1	0.74
DQB1*05:18:01	1	0.35	2	1.20	DPB1*104:01:01:13	0	0.00	1	0.74
DQB1*05:239	3	1.04	0	0.00	DPB1*105:01:01:02	5	1.95	2	1.48
DQB1*06:01:01:01	33	11.42	16	9.58	DPB1*13:01:01:01	1	0.39	3	2.22
DQB1*06:02:01:01	10	3.46	4	2.40	DPB1*13:01:01:02	11	8.56	18	5.19
DQB1*06:03:01:01	2	0.69	1	0.60	DPB1*13:01:01:05	26	10.12	12	8.89
DQB1*06:09:01:01	1	0.35	2	1.20	DPB1*1315:01	2	0.78	0	0.00
Alleles analyzed	289	100.00	167	100.00	DPB1*135:01:01:01	3	1.17	0	0.00
					DPB1*14:01:01:01	14	5.45	6	4.44
					DPB1*14:01:01:05	3	1.17	1	0.74
					DPB1*15:01:01:01	1	0.39	0	0.00
					DPB1*17:01:01:01	3	1.17	4	2.96
					DPB1*19:01:01:01	1	0.39	0	0.00
					DPB1*21:01:01	11	4.28	1	0.74
					DPB1*26:01:02:01	1	0.39	1	0.74
					DPB1*28:01:01:01	1	0.39	0	0.00
					DPB1*296:01	6	2.33	1	0.74
					DPB1*31:01:01:01	2	0.78	5	3.70
					DPB1*38:01	1	0.39	0	0.00
					DPB1*45:01	1	0.39	0	0.00
					DPB1*48:01	0	0.00	1	0.74
					DPB1*744:01	0	0.00	1	0.74
					Alleles analyzed	246	100.00	146	100.00

### Associations of HLA alleles and anti-rHuEPO-induced PRCA

Table 5 shows the significant HLA alleles associated with anti-rHuEPO-induced PRCA. In the no covariate analysis models (Models 1 and 2), HLA-B*46:01:01:01, HLA-DRB1*09:01:02:01, and HLA-DQB1*03:03:02:02 were highly related to anti-rHuEPO-induced PRCA (OR (95%CI), p-values for Model 1: Additive: HLA-B*46:01:01:01 = 4.58 (1.55–13.51), p = 0.006, HLA-DRB1*09:01:02:01 = 3.99 (1.28–12.49), p = 0.017, and HLA-DQB1*03:03:02:02 = 4.70 (1.14–19.41), p = 0.033; Model 2: Dominant: HLA-B*46:01:01:01 = 4.63 (1.56–13.75), p = 0.006, HLA-DRB1*09:01:02:01 = 4.50 (1.32–15.40), p = 0.016, and HLA-DQB1*03:03:02:02 = 5.17 (1.18–22.74), p = 0.030). However, HLA-A*02:01:01:01, HLA-A*24:07:01:01, HLA-B*38:02:01:01, and HLA-C*15:02:01:01, HLA-DRB1*04:05:01:01, and HLA-DPB1*04:02:01:02 showed only slightly increased ORs in Models 1 and 2.

**Table 5:** Significant HLA alleles associated with anti-rHuEPO-induced PRCA.

Models	Allele	Observed p-value	Permutation p-value	Odds ratio with 95% confidence interval
Model 1: additive	A*02:01:01:01	0.018	0.004	0.19 (0.05–0.75)
	B*38:02:01:01	0.019	0.005	0.17 (0.04–0.75)
	B*46:01:01:01	0.006	0.004	4.58 (1.55–13.51)
	C*15:02:01:01	0.037	0.017	0.28 (0.09–0.93)
	DRB1*04:05:01:01	0.047	0.022	0.19 (0.04–0.98)
	DRB1*09:01:02:01	0.017	0.012	3.99 (1.28–12.49)
	DQB1*03:03:02:02	0.033	0.016	4.70 (1.14–19.42)
Model 2: dominant	A*02:01:01:01	0.018	0.005	0.17 (0.04–0.74)
	A*24:07:01:01	0.058	0.028	0.33 (0.10–1.04)
	B*38:02:01:01	0.019	0.005	0.17 (0.04–0.75)
	B*46:01:01:01	0.006	0.004	4.63 (1.56–13.75)
	C*15:02:01:01	0.037	0.016	0.28 (0.09–0.93)
	DRB1*04:05:01:01	0.045	0.031	0.17 (0.03–0.96)
	DRB1*09:01:02:01	0.016	0.012	4.50 (1.32–15.40)
	DQB1*03:03:02:02	0.030	0.018	5.17 (1.18–22.74)
	DPB1*04:02:01:02	0.045	0.031	0.17 (0.03–0.96)
Model 3: additive adjusted with types of rHuEPO	A*02:01:01:01	0.035	0.012	0.21 (0.05–0.90)
	B*38:02:01:01	0.018	0.007	0.17 (0.04–0.74)
	B*46:01:01:01	0.006	0.004	5.72 (1.67–19.67)
	C*15:02:01:01	0.010	0.005	0.17 (0.05–0.65)
	DRB1*09:01:02:01	0.035	0.028	3.42 (1.09–10.74)
Model 4: dominant adjusted with types of rHuEPO	A*02:01:01:01	0.040	0.016	0.20 (0.04–0.93)
	B*38:02:01:01	0.018	0.007	0.17 (0.04–0.74)
	B*46:01:01:01	0.005	0.004	5.81 (1.68–20.09)
	C*15:02:01:01	0.010	0.005	0.17 (0.05–0.65)
	DRB1*09:01:02:01	0.038	0.032	3.75 (1.08–13.07)

*rHuEPO* recombinant human erythropoietin.

Additive: homozygous for the tested allele was coded 2, heterozygous was coded 1, and other alleles were coded 0.

Dominant: homozygous and heterozygous for the tested allele were coded 1, and other alleles were coded 0.

Types of rHuEPO: innovator and biocopy.

When data were adjusted by the types of rHuEPO as a covariate (Models 3 and 4, Table 5), some HLA alleles lost significance, leaving anti-rHuEPO-induced PRCA highly associated with the presence of HLA-B*46:01:01:01 and HLA-DRB1*09:01:02:01 (OR (95%CI), p-values for Model 3: Additive adjusted with types of rHuEPO: HLA-B*46:01:01:01 = 5.72 (1.67–19.67), p = 0.006 and HLA-DRB1*09:01:02:01 = 3.42 (1.09–10.74), p = 0.035; Model 4: Dominant adjusted for types of rHuEPO: HLA-B*46:01:01:01 = 5.81 (1.68–20.09), p = 0.006 and HLA-DRB1*09:01:02:01 = 3.75 (1.08–13.07), p = 0.038). Multiple logistic regression analyses for the interaction between each pair of HLA-B*46:01:01:01, HLA-DRB1*09:01:02:01, and HLA-DQB1*03:03:02:02 were performed under additive and dominant models with no interaction observed (Supplementary Table 1).

HLA-A*02:01:01:01, HLA-B*38:02:01:01, and HLA-C*15:02:01:01 were also significantly associated with anti-rHuEPO-induced PRCA in Models 3 and 4 (Table 5), but with very low OR (OR (95% CI), p-values for HLA-A*02:01:01:01 = 0.21 (0.05–0.90), p = 0.035 in Model 3 and 0.20 (0.04–0.93), p = 0.040 in Model 4; HLA-B*38:02:01:01 = 0.17 (0.04–0.74), p = 0.018 in Models 3 and 4, and HLA-C*15:02:01:01 = 0.17 (0.05–0.65), p = 0.010 in Models 3 and 4).

### Associations of HLA haplotypes and anti-rHuEPO-induced PRCA

**Figure 1:**
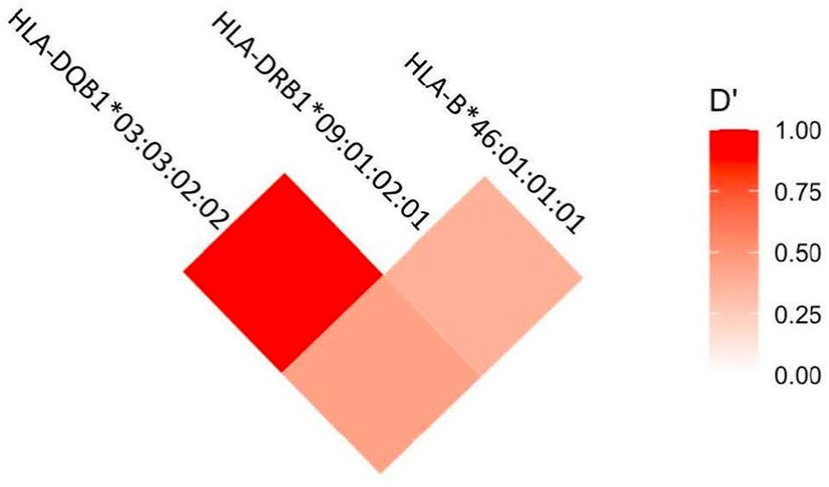
Heatmap of HLA-B*46:01:01:01-DRB1*09:01:02:01-DQB1*03:03:02:02. Linkage disequilibrium (LD) expressed in terms of the D’ measure. Color-bar represents the range of D’ (0–1). Bright red colors represent lower values of D’, while dark red colours demonstrate high haplotype LD.

When 6-locus haplotypes (HLA-A, HLA-B, HLA-C, HLA-DRB1, HLA-DQB1, and HLA-DPB1) were analyzed, 111 HLA haplotypes were estimated (Supplementary Table 2). We focus our analysis using significant HLA alleles from logistic regression analyses as haplotype (HLA-B*46:01:01:01-HLA-DRB1*09:01:02:01-HLA-DQB1*03:03:02:02), no significant association was observed between cases and controls. When we looked into details, we found that this focused 3-locus haplotype was found only in 12 cases (Table 6) and none in controls. The linkage disequilibrium analysis of HLA-B*46:01:01:01, HLA-DRB1*09:01:02:01, and HLA-DQB1*03:03:02:02 showed significant relationships where HLA-DRB1 and HLA-DQB1 were highly linked (D' = 0.907152, Corr = 0.746613, p = 0.00, Table 7, Fig. 1).

**Table 6:** Cases with HLA-B*46:01:01:01-HLA-DRB1*09:01:02:01-HLA-DQB1*03:03:02:02 haplotype.

Case no.	HLA-B	HLA-DRB1	HLA-DQB1
04	B*46:01:01:01/ B*27:05:02:01	DRB1*09:01:02:01/ DRB1*09:01:02:01	DQB1*03:03:02:02/ DQB1*03:02:01:01
27	B*46:01:01:01/ B*15:32:01	DRB1*09:01:02:01/ DRB1*15:02:02:01	DQB1*03:03:02:02/ DQB1*06:01:01:01
29	B*46:01:01:01/ B*46:88	DRB1*09:01:02:01/ DRB1*14:51:01:01	DQB1*03:03:02:02/ DQB1*05:02:01:01
33	B*46:01:01:01/ B*18:01:01:02	DRB1*09:01:02:01/ DRB1*16:02:01:06	DQB1*03:03:02:02/ DQB1*05:02:01:01
53	B*46:01:01:01/ B*39:09:01:01	DRB1*09:01:02:01/ DRB1*09:01:02:01	DQB1*03:03:02:02/ DQB1*03:02:01:01
56	B*46:01:01:01/ B*15:01:01:01	DRB1*09:01:02:01/ DRB1*09:01:02:01	DQB1*03:02:01:01/ DQB1*03:03:02:02
76	B*46:01:01:01/ B*07:02:01:01	DRB1*09:01:02:01/ DRB1*14:04:01:01	DQB1*03:03:02:02/ DQB1*05:03:01:01
97	B*46:01:01:01/ B*39:01:01:03	DRB1*09:01:02:01/ DRB1*12:02:01:01	DQB1*03:03:02:02/ DQB1*03:01:01:07
107	B*46:01:01:01/ B*56:01:01:03	DRB1*09:01:02:01/ DRB1*09:01:02:01	DQB1*03:03:02:02/ DQB1*04:01:01:03
119	B*46:01:01:01/ B*58:01:01:03	DRB1*09:01:02:01/ DRB1*03:01:01:01	DQB1*03:03:02:02/ DQB1*03:03:02:02
123	B*46:01:01:01/ B*15:25:01:01	DRB1*09:01:02:01/ DRB1*04:03:02	DQB1*03:03:02:02/ DQB1*05:01:24:01
125	B*46:01:01:01/ B*15:32:01	DRB1*09:01:02:01/ DRB1*15:02:02:01	DQB1*03:03:02:02/ DQB1*06:01:01:01

**Table 7:** Level of significant of the linkage disequilibrium between each pair of HLA loci.

Locus pair	D'	Corr	p-value
HLA-B: HLA-DRB1	0.372536	0.322753	1.73 × 10^–9^
HLA-B: HLA-DQB1	0.443488	0.316227	4.97 × 10^–9^
HLA-DRB1: HLA-DQB1	0.907152	0.746613	0.00

### rHuEPO-HLA molecular docking analysis

**Figure 2:**
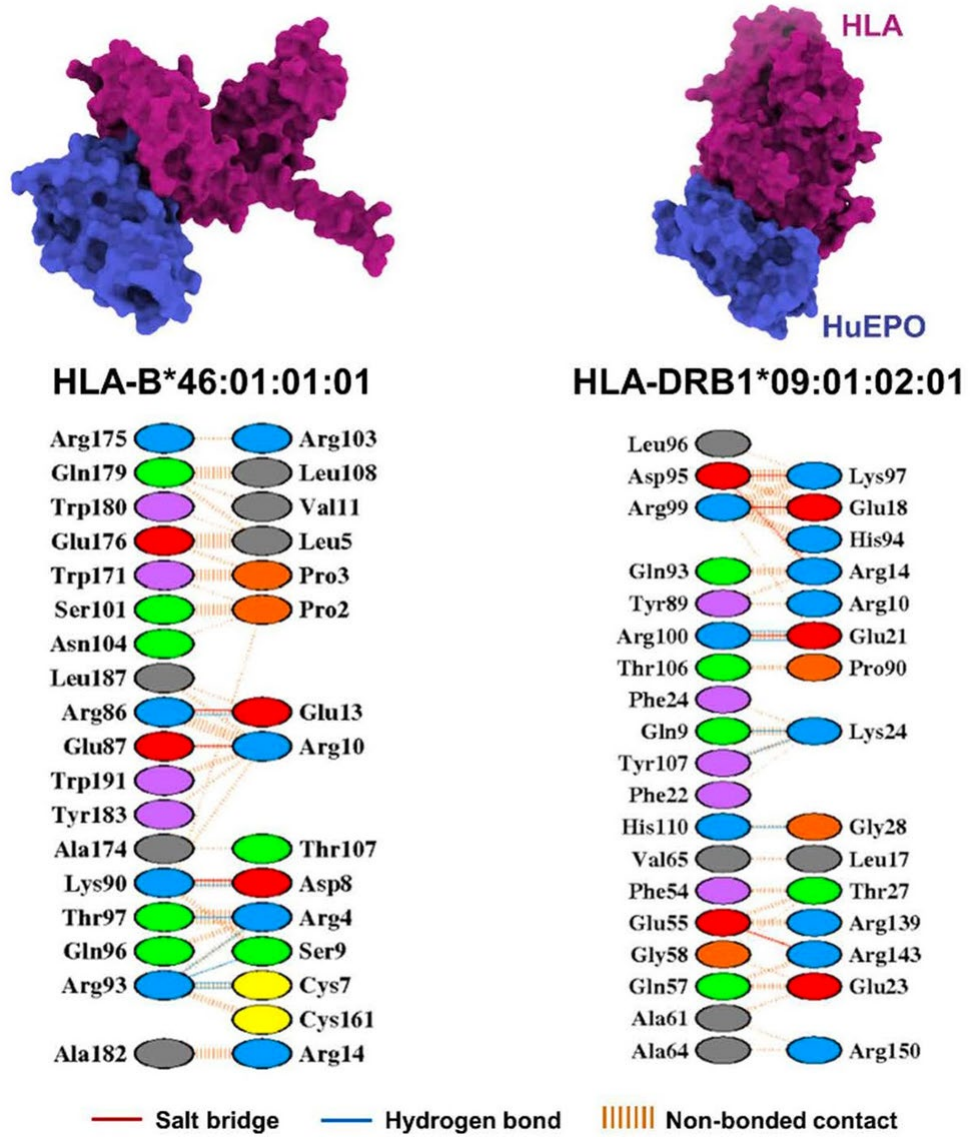
The binding patterns and key residues of the structure of human erythropoietin complexed with HLA-B*46:01:01:01 and HLA-DRB1*09:01:02:01 analyzed through salt bridge interactions, hydrogen bondings, and non-bonded contacts.

A molecular docking study was performed to access the binding of rHuEPO to the HLA alleles using the HDOCK webserver^22^. The docking results of the rHuEPO-HLA complexes are presented in Table 8. In particular, the HLA-B*46:01:01:01 and HLA-DRB1*09:01:02:01 alelles exhibited a superior HDOCK score (∆*G*_bind_) of −160.79 and −165.47 kcal/mol, respectively, outperforming the others, including HLA-DQB1*03:03:02:02 (−153.52 kcal/mol). The protein–protein interactions of rHuEPO in complex with HLA-B*46:01:01:01 and HLA-DRB1*09:01:02:01 included salt bridges, hydrogen bonding, and non-bonded contacts. Furthermore, the binding patterns and critical residues of these two focused systems were elucidated by analysing these interactions, as shown in Fig. 2.

**Table 8:** Binding affinity presented by ∆*G*_bind_, salt bridge and hydrogen bonding interactions, analyzed in the molecular docking study of human erythropoietin against different genetic variations in HLA.

Human leukocyte antigen	∆*G*_bind_ (kcal/mol)	# Salt bridge	# Hydrogen bond
HLA-A*02:01:01:01	−156.29	2	3
HLA-B*38:02:01:01	−156.55	3	2
HLA-B*46:01:01:01	−160.79	3	7
HLA-C*15:02:01:01	−150.29	2	3
HLA-DRB1*09:01:02:01	−165.47	5	5
HLA-DQB1*03:03:02:02	−153.52	4	5

## Discussion

We successfully conducted a case–control study to identify genetic variation in HLA as a significant risk factor for anti-rHuEPO-induced PRCA in patients with CKD using the highest accuracy long-read sequencing technique. Regardless of the types of rHuEPO, CKD patients with HLA-B*46:01:01:01 or HLA-DRB1*09:01:02:01 had a three to sixfold increased risk of developing anti-rHuEPO-induced PRCA.

HLA associations of HLA with antibody-positive PRCA have been reported in other studies^2–4,7^. Furthermore, previous data in Thai patients with CKD (22 cases) have strongly suggested the association of HLA-DRB1*09 and anti-rHuEPO-induced PRCA^4^. Here, our study was carried out in a larger cohort size (157 cases, not including 22 cases in the previous study^4^), with the highest resolution of the HLA genotyping platform, confirming the risk of anti-rHuEPO-induced PRCA in patients with HLA-DRB1*09 and, more specifically, HLA-DRB1*09:01:02:01. Our study also adds the new information that HLA-B*46:01:01:01 is also associated with a very high risk of anti-rHuEPO-induced PRCA.

A report in Caucasians (24 cases) also showed a significant association between HLA-DRB1*09 and antibody-positive PRCA^3^. However, the allele frequency of HLA-DRB1*09 alleles in Caucasians was lower than the data observed in our Thai cohort (cases/controls = 12.70/5.92% (Table 3) and 9.6/ 0.7% for this study and Fijal et al.^3^, respectively). Differences in HLA-DRB1*09 allelles in the background of Thai and Caucasian populations could partly explain the low incidence of anti-rHuEPO-induced PRCA in western countries. A study in Chinese patients suggested the association of HLA-DRB1*12:02 with PRCA^7^. Here, HLA-DRB1*12:02:01:01 was found highly in our cases and controls (16.94% vs 18.34%, Table 3) but no association with anti-rHuEPO-induced PRCA was shown in our analyses. Furthermore, other HLA alleles (HLA-A*25, HLA-B*53, HLA-C*12, HLA-DRB1*04, HLA-DQB1*03, and HLA-DQB1*06) were also identified as risk factors for PRCA^3^. It should be noted that in our data there were no HLA-A*25 and HLA-B*53 (Table 2).

The mechanism of PRCA in patients with CKD that are correlated with HLA needs to be clearly defined. However, the immunogenic antibody activated by rHuEPO was related to T cell activation^23,24^. HLA Class II, including HLA-DR, HLA-DQ, and HLA-DP, are expressed primarily in antigen-presenting cells that uptake, process, and present the antigen as a peptide epitope in naive T cells with a Class II molecule of the major histocompatibility complex (MHC) on its surface. Binding between the MHC class II epitope complex and the T cell receptor activates the T cell to release cytokines to trigger the differentiation of B cells into plasma cells. Plasma cells then secrete antibodies against the corresponding epitope^23^. Our study revealed that HLA-B*46:01:01:01 and HLA-DRB1*09:01:02:01 alleles demonstrated strong interactions with crucial residues of rHuEPO, such as R103, T107, L108, R143, and R150 (Table 8, Fig. 2). These results are consistent with those reported in a previous study^25^. Consequently, the favorable interactions observed between rHuEPO and the mentioned HLAs could potentially stimulate the immune defense system.

To our knowledge, our data are the largest cohort of patients with CKD who suffer from rHuEPO-induced PRCA. Furthermore, our study is the first study to display the frequency of the HLA alleles and the HLA haplotypes in the Thai population with a highly accurate long-read SMRT sequencing technique. SMRT sequencing differs from other next-generation sequencing tools by providing longer reads and higher accuracy and is ideal for situations where high precision, high recall, and full-length data of the interest gene, for example, the HLA gene, are required^26^. The eight digits of HLA alleles can be obtained with long-read SMRT sequencing, allowing full display of the frequency of HLA alleles in the population. Previous reports on HLA allele frequency distribution reports in the general Thai population were performed with lower resolution techniques that captured only HLA antigens (n = 16,807)^27^ or four digits of HLA (n = 470)^28^. When we analyzed our data using only two or four digits of HLA, the frequency distribution of the HLA allele was consistent with previous data^27,28^. Therefore, we are confident that the associations of HLA and PRCA presented in this study are truly valid. Importantly, with a highly accurate long-read SMRT sequencing technique, we were able to identify 3, 5, 9, and 4 subtypes of HLA-C*12, HLA-DRB1*04, HLA-DQB1*03, and HLA-DQB1*06 in our study (Tables 2, 3 and 4).

However, this study has limitations. The study was carried out in a case–control design only in the Thai population, so data should be interpreted for other ethnicities with caution. Furthermore, the observed association may not imply causal relationships. As a retrospective study, data on the immune status of patients were unavailable. The analyses were corrected for the originality of rHuEPO, not for all the branding of the rHuEPO biocopy products. Therefore, the variability in different rHuEPO biocopy products must be considered in a further study, as previous reports show the variability in their quality. In fact, a substantial amount of various protein fragments and different stabilizers used in the products were found in the rHuEPO biocopy products, and this could be a source of increased immunogenicity related to the rHuEPO biocopy^19,21^. There was no data on the transport and preservation technique. However, patients received rHuEPO from qualified nephrology clinics in hospitals; therefore, we believe that the transport and preservation technique was of a similar standard. Anti-rHuEPO-induced PRCA was reported to be not related to age^20^; therefore, we did not include age in our models. Although HLA-B*46:01:01:01-HLA-DRB1*09:01:02:01-HLA-DQB1*03:03:02:02 showed significant linkage disequilibrium, 3-locus haplotype was found only in cases (Table 6) and none in controls. Therefore, the odd ratios of this 3-locus haplotype between cases and controls cannot be determined. The susceptible HLA alleles identified in our study were found in 45% for HLA-B*46:01:01:01 and 12.7% for HLA-DRB1*09:01:02:01 of the PRCA cases. Therefore, it could be that other unidentified factors contributed to the susceptibility of anti-rHuEPO-induced PRCA.

In conclusion, with the highest accuracy, the long-read SMRT sequencing technique, HLA-B*46:01:01:01 and HLA-DRB1*09:01:02:01 markedly increase the risk of anti-rHuEPO-induced PRCA in patients with CKD. This suggests the role of pharmacogenetic test of these HLA alleles to help clinicians closely monitor their patients for anti-rHuEPO-induced PRCA. However, since the cost of HLA sequencing is still relatively high, more studies are needed to test the cost-effectiveness of pharmacogenetic testing, especially in resource-limited countries.

## Methods

The study was a retrospective case–control study, carried out according to the Declaration of Helsinki of the World Medical Association, following the International Conference on Harmonization Guidelines for Good Clinical Practice. The Institutional Review Board of the Faculty of Medicine, Chulalongkorn University, Bangkok, Thailand approved the study protocol (IRB number 807/62). All participants provided their written informed consent for their participation prior to the start of the study.

### Participants

Adult CKD patients with anti-rHuEpo-induced PRCA according to the database of severe adverse drug reactions, Division of Nephrology, Department of Medicine, King Chulalongkorn Memorial Hospital, Thailand, were enrolled. As our center is the only center in Thailand that provides an anti-rHuEPO antibody radioimmunoprecipitation test for the diagnosis of PRCA, samples of suspected PRCA were sent to us from nephrology clinics in hospitals throughout the country. All anti-rHuEPO-induced PRCA developed a sudden onset of loss of rHuEPO efficacy, had a low reticulocyte count (< 10 × 10^9^/L), positive anti-rHuEPO antibody by radioimmunoprecipitation^29,30^, and bone marrow biopsy showed normocellularity in the absence of erythroid precursor (< 5% of erythroblast in bone marrow) with normal myeloid and megakaryocytic lineages.

Controls were recruited from the database of an outpatient nephrology clinic at King Chulalongkorn Memorial Hospital, Thailand. Controls were nondialysis CKD patients treated with rHuEPO for more than two years without developing anti-rHuEPO-induced PRCA, as evidence suggested that anti-rHuEPO-induced PRCA occurred during 9–24 months of exposure to EPO^4,13,14^. All cases and controls were recruited during the same period of time (2019–2022). Patients with CKD with an unexplained cause of PRCA, patients treated with immunosuppressive agents, or patients who underwent organ transplantation were excluded.

The rHuEPO innovator products were defined as the first drugs created containing their specific active ingredient to receive approval for use^31,32^. The rHuEPO biosimilar products were defined according to the guidelines of the United States Food & Drug Administration^31^ and/or the European Medicines Agency^32^. Generic rHuEPO products that did not meet those criteria were considered rHuEPO biocopy products.

### Genomic DNA extraction and quality control assessment

According to the manufacturer's instructions, human genomic DNA (gDNA) was extracted from whole EDTA blood using a QIAamp DNA mini kit (QIAGEN Inc. GmbH, Hilden, Germany). DNA concentration of all samples was measured using a Qubit Fluorometer and Qubit dsDNA HS kit (Thermo Fisher Scientific, Waltham, MA, USA). Furthermore, the integrity and purity of the samples were also assessed with agarose gel electrophoresis and a NanoDrop One UV–Vis spectrophotometer (Thermo Fisher Scientific, Waltham, MA, USA). Only high quality samples were used for the subsequent analysis (A260/A280 = 1.7–2.0, tight band of high molecular weight gDNA and no strong smear band below 20 kb in size).

### HLA genes amplification

HLA Class I (HLA-A, HLA-B, and HLA-C) and HLA Class II (HLA-DRB1, HLA-DRB3, HLA-DRB4, HLA-DQB1, and HLA-DPB1) genes were amplified from 100 ng gDNA samples using a multiplex polymerase chain reaction (PCR), (NXType NGS Amplification Kit, One Lambda Inc., West Hills, CA, USA). The PCR amplification conditions included an initial denature at 94 °C for 2 min, followed by heating at 98 °C for 10 s and 69 °C for 3 min, repeated for 30 cycles. All obtained PCR products were purified with an AMPure^®^ PB kit (Pacific Biosciences Inc., Menlo Park, CA, USA). The accurate size of the purified HLA amplicons was verified with the Agilent DNA 12,000 kit (Agilent Technologies, Inc., Santa Clara, CA, USA) and the concentration was measured using the Qubit Fluorometer and the Qubit dsDNA HS kit (Thermo Fisher Scientific, Waltham, MA, USA). Subsequently, all qualified samples were diluted to 10 ng/µl for HLA Class I and 20 ng/µl for HLA Class II.

### Long-read HLA sequencing and HLA genotyping

The preparation of HLA single-molecule, real-time bell (SMRTbell) libraries was carried out with a 96-SMRTbell barcode adapter from the complete prep kit. Briefly, amplicon libraries were prepared using PacBio barcode adapters for multiplex single-molecule real-time sequencing (SMRT), (PN 100-538-700-03, Pacific Biosciences Inc., Menlo Park, CA, USA). The HLA amplicons were prepared as 96 samples per library. The total input of DNA for each library was 4.8 µg, prepared from the equimolar pooling of each sample's HLA Class I and Class II. The HLA libraries were sequenced in the PacBio Sequel System (Sequencing primer V3, movie time of 10 h per SMRT cell, Pacific Biosciences Inc., Menlo Park, CA, USA). Sequencing data were demultiplexed according to the adapter sequence, followed by a long-amplicon analysis using the SMRT Analysis software version 9.0 to obtain consensus sequences (FASTQ file).

HLA genotyping was carried out using GenDx NGSengine® software (Genome Diagnostic B.V., Utrecht, The Netherlands) based on the IMGT/HLA database version 3.52.0 (http://www.ebi.ac.uk/imgt/hla). Linkage disequilibrium analysis of significant HLA alleles (HLA-B*46:01:01:01, HLA-DRB1*09:01:02:01, and HLA-DQB1*03:03:02:02) was calculated using the R package for genetic analysis (https://rdrr.io/cran/genetics/man/LD.html) and was also visualized by ggplot2 (https://cran.r-project.org/web/packages/ggplot2/index.html).

### In silico study of the binding of rHuEPO to HLA

Molecular docking investigation was used to investigate the interaction characteristics of the rHuEPO with models of HLA-A*02:01:01:01, HLA-B*38:02:01:01, HLA-B*46:01:01:01, HLA-C*15:02:01:01, HLA-DRB1*09:01:02:01, and HLA-DQB1*03:03:02:02 alelles. The three-dimensional (3D) structure of rHuEPO was obtained from the protein data bank (PDB)^33^, while the structural geometries of HLA were received from the FASTQ files of this study. First, multiple alignment with fast fourier transform (MAFFT) program^34^ was applied to generate a consensus sequence. Standard nucleotide BLAST (BLASTN)^35^ was used to recheck the accuracy of the sequence. The verified consensus sequences were then used for gene annotation and converted into protein sequences through the genome bioinformatics research laboratory (Geneid WEB server)^36^ and the Expasy webserver^37^, respectively. HLA 3D structures were predicted using AlphaFold2 software^38^.

To construct the HLA structures for the docking study, the predicted HLA structures were superimposed on the crystal structure of Human Leukocyte Antigen F (HLA-F), which presents peptides and regulates immunity through interactions with NK cell receptors (PDB: 5KNM^39^) for HLA Class I, and the crystal structure of HLA-DRB1 in complex with Type II collagen peptide (PDB: 6BIN^40^) for HLA Class II using the University of California at San Francisco (UCSF) Chimera package^41^. The ionized states of HLA and rHuEPO were configured at pH 7.0 using PROPKA3.1^42^. Consequently, rHuEPO was docked to HLA pocket sites using HDOCK server^22^. The 3D and 2D binding interactions of rHuEPO-HLA were visualized using the UCSF Chimera package^41^ and the PDBsum web server^43^, respectively.

### Statistical analysis

Data were collected and analyzed using the SPSS version 29 and the R program. Continuous and catergorical data were presented in mean (range) or n (%), respectively, unless otherwise indicated. The distribution of the continuous baseline data was compared using the two-sample Kolmogorov–Smirnov test. The frequency of each HLA allele in cases and controls was counted directly and presented as n (%). The percentage was calculated as [the number of that allele detected/ the total number of alleles analyzed] × 100. The frequency of HLA haplotypes was estimated using the haplo.em function, which computed maximum likelihood estimates of haplotype probabilities in the haplo.stats R package, and was presented as percentage. Six-locus HLA haplotypes (A–B–C–DRB1–DQB1–DPB1) were estimated from the data through an expectation–maximization (EM).

Univariate logistic regression with the general linear model function in the R program was used. The logit link function was performed under additive and dominant models to test for associations between each allele and the phenotype. For the univariate analysis, two models were analyzed [Model 1: Additive (homozygous for the tested allele was coded 2, heterozygous was coded 1, and other alleles were coded 0) and Model 2: Dominant (homozygous and heterozygous for the tested allele were coded 1, and other alleles were coded 0)].

Multivariate logistic regression analyses were performed for additive and dominant models adjusted by types of rHuEPO (innovator and biocopy), [Model 3: Additive model adjusted with types of rHuEPO and Model 4: Dominant model adjusted with the types of rHuEPO]. The odds ratio (OR) was calculated and presented with a 95% confidence interval (95%CI). The permutation test (100,000 permutations) calculated the p-values for multiple tests. Significant HLA alleles derived from multivariate logistic regression analyses were further analyzed for their interactions using multiple logistic regression analyses. For all analyses, significance was set at a p-value < 0.05.

### Supplementary Information

Supplementary Tables.

## Data Availability

The data sets used and/or analyzed during the current study are available from the corresponding author on reasonable request.
