# Supplementary material for: HLA-B*46:01:01:01 and HLA-DRB1*09:01:02:01 are associated with anti-rHuEPO-induced pure red cell aplasia
Source: Sci Rep. 2023 Dec 20;13:22759. doi: 10.1038/s41598-023-50211-3 (PMC10733298; doi:10.1038/s41598-023-50211-3)
Supplement: Supplementary file 1 — Supplementary Tables. [file 41598_2023_50211_MOESM1_ESM.docx]

**Supplementary Tables**

**HLA-B*46:01:01:01 and HLA-DRB1*09:01:02:01 are associated with anti-rHuEPO-induced pure red cell aplasia**

Thitima Benjachat Suttichet^1^, Monpat Chamnanphon^1^, Monnat Pongpanich^2, 3^, Sarun Chokyakorn^4^, Pawinee Kupatawintu^5^, Chalurmpon Srichomthong^6, 7^, Wanna Chetruengchai^6, 7^, Hathaichanok Chuntakaruk^8, 9^, Thanyada Rungrotmongkol^8, 9^, Pajaree Chariyavilaskul^1, 4, *^, Vorasuk Shotelersuk^6, 7^, Kearkiat Praditpornsilpa^10^

^1^Center of Excellence in Clinical Pharmacokinetics and Pharmacogenomics, Faculty of Medicine, Chulalongkorn University, Bangkok, Thailand.

^2^Department of Mathematics and Computer Science, Faculty of Science, Chulalongkorn University, Bangkok, Thailand.

^3^Faculty of Science, Omics Sciences and Bioinformatics Center, Chulalongkorn University, Bangkok, Thailand.

^4^Department of Pharmacology, Faculty of Medicine, Chulalongkorn University, Bangkok, Thailand.

^5^National Blood Center, Thai Red Cross Society, Bangkok, Thailand.

^6^Center of Excellence for Medical Genomics, Department of Pediatrics, Faculty of Medicine, Chulalongkorn University, Bangkok, Thailand.

^7^Excellence Center for Genomics and Precision Medicine, King Chulalongkorn Memorial Hospital, the Thai Red Cross Society, Bangkok, Thailand.

^8^Program in Bioinformatics and Computational Biology, Graduate School, Chulalongkorn University, Bangkok, Thailand.

^9^Center of Excellence in Structural and Computational Biology, Department of Biochemistry, Faculty of Science, Chulalongkorn University, Bangkok, Thailand.

^10^Division of Nephrology, Department of Medicine, Faculty of Medicine, Chulalongkorn University, Bangkok, Thailand.

***Correspondence**

Pajaree Chariyavilaskul, Department of Pharmacology, Faculty of Medicine, Chulalongkorn University, Bangkok, Thailand

Email: [pajaree.l@chula.ac.th](mailto:pajaree.l@chula.ac.th), Tel: 006622564481 ext. 3020

**Keywords:** pure red cell aplasia, recombinant human erythropoietin, chronic kidney disease, HLA, long-read sequencing

**Running title:** HLA-B*46:01:01:01 & DRB1*09:01:02:01 and PRCA

**Supplementary Table 1 Multiple logistic regression analysis for the interaction between each pair of HLA loci.**

| **Model** | **Variables** | **Estimate** | **Standard error** | **p-value** |
| --- | --- | --- | --- | --- |
| **Additive models** | | | | |
| **Interaction between**  **HLA-B*46:01:01:01**  **and HLA-DRB1*09:01:02:01** | (Intercept) | 0.701 | 0.199 | 0.00044 |
|  | HLA-B*46:01:01:01 | 1.004 | 0.579 | 0.08291 |
|  | HLA-DRB1*09:01:02:01 | 0.104 | 0.877 | 0.90540 |
|  | HLA-DQB1*03:03:02:02 | 0.301 | 0.999 | 0.76364 |
|  | HLA-B*46:01:01:01/ HLA-DRB1*09:01:02:01 | 15.306 | 1289.760 | 0.99053 |
| **Interaction between**  **HLA-B*46:01:01:01**  **and HLA-DQB1*03:03:02:02** | (Intercept) | 0.697 | 0.199 | 0.00046 |
|  | HLA-B*46:01:01:01 | 1.045 | 0.576 | 0.06991 |
|  | HLA-DRB1*09:01:02:01 | 0.202 | 0.854 | 0.81325 |
|  | HLA-DQB1*03:03:02:02 | 0.221 | 0.978 | 0.82105 |
|  | HLA-B*46:01:01:01/ HLA-DQB1*03:03:02:02 | 14.876 | 958.386 | 0.98762 |
| **Interaction between**  **HLA-DRB1*09:01:02:01**  **and HLA-DQB1*03:03:02:02** | (Intercept) | 0.679 | 0.198 | 0.00061 |
|  | HLA-B*46:01:01:01 | 1.257 | 0.573 | 0.02818 |
|  | HLA-DRB1*09:01:02:01 | 0.236 | 0.976 | 0.80902 |
|  | HLA-DQB1*03:03:02:02 | -0.009 | 1.566 | 0.99525 |
|  | HLA-DRB1*09:01:02:01/ HLA-DQB1*03:03:02:02 | 0.515 | 1.511 | 0.73348 |
| **Dominant models** |  |  |  |  |
| **Interaction between**  **HLA-B*46:01:01:01**  **and HLA-DRB1*09:01:02:01** | (Intercept) | 0.718 | 0.201 | 0.00035 |
|  | HLA-B*46:01:01:01 | 0.987 | 0.579 | 0.08867 |
|  | HLA-DRB1*09:01:02:01 | 0.024 | 0.990 | 0.98060 |
|  | HLA-DQB1*03:03:02:02 | 0.177 | 1.184 | 0.88088 |
|  | HLA-B*46:01:01:01/ HLA-DRB1*09:01:02:01 | 15.670 | 1057.000 | 0.98817 |
| **Interaction between**  **HLA-B*46:01:01:01**  **and HLA-DQB1*03:03:02:02** | (Intercept) | 0.714 | 0.200 | 0.00036 |
|  | HLA-B*46:01:01:01 | 1.030 | 0.577 | 0.07417 |
|  | HLA-DRB1*09:01:02:01 | 0.154 | 0.954 | 0.87193 |
|  | HLA-DQB1*03:03:02:02 | 0.071 | 1.161 | 0.95116 |
|  | HLA-B*46:01:01:01/ HLA-DQB1*03:03:02:02 | 15.600 | 1097.000 | 0.98866 |
| **Interaction between**  **HLA-DRB1*09:01:02:01**  **and HLA-DQB1*03:03:02:02** | (Intercept) | 0.671 | 0.198 | 0.00070 |
|  | HLA-B*46:01:01:01 | 1.322 | 0.581 | 0.02300 |
|  | HLA-DRB1*09:01:02:01 | 0.529 | 1.148 | 0.64484 |
|  | HLA-DQB1*03:03:02:02 | 13.895 | 882.743 | 0.98744 |
|  | HLA-DRB1*09:01:02:01/ HLA-DQB1*03:03:02:02 | -13.702 | 882.745 | 0.98762 |

**Supplementary Table 2 Estimated haplotype frequency of 6-locus HLA haplotype (HLA-A, HLA-B, HLA-C, HLA-DRB1, HLA-DQB1 and HLA-DPB1.**

| **Haplotype number** | **HLA-A** | **HLA-B** | **HLA-C** | **HLA-DRB1** | **HLA-DQB1** | **HLA-DPB1** | **Haplotype frequency (%)** |
| --- | --- | --- | --- | --- | --- | --- | --- |
| 1 | 01:01:01:01 | 37:01:01:01 | 07:06:01:01 | 10:01:01:01 | 03:01:01:02 | 26:01:02:01 | 0.79 |
| 2 | 01:01:01:01 | 52:01:01:01 | 06:02:01:01 | 07:01:01:01 | 05:03:01:01 | 09:01:01:01 | 0.79 |
| 3 | 01:01:01:01 | 57:01:01:01 | 03:03:01:01 | 12:01:01:01 | 03:01:01:01 | 09:01:01:01 | 0.79 |
| 4 | 02:01:01:01 | 58:01:01:01 | 03:02:02:01 | 03:01:01:01 | 06:01:01:01 | 05:01:01:01 | 0.79 |
| 5 | 02:03:01:01 | 07:06:01:01 | 07:02:01:01 | 15:02:01:02 | 05:01:24:01 | 02:01:02:01 | 0.79 |
| 6 | 02:03:01:01 | 18:01:01:02 | 03:03:01:01 | 15:02:01:02 | 05:01:24:01 | 14:01:01:01 | 0.79 |
| 7 | 02:03:01:01 | 38:02:01:01 | 07:02:01:01 | 12:02:01:01 | 03:01:01:07 | 04:01:01:01 | 0.79 |
| 8 | 02:03:01:01 | 38:02:01:01 | 07:02:01:01 | 15:02:01:02 | 05:01:24:01 | 13:01:01:05 | 0.79 |
| 9 | 02:03:01:01 | 39:09:01:01 | 01:02:01:01 | 14:54:01:01 | 05:02:01:01 | 03:01:01:01 | 0.79 |
| 10 | 02:03:01:01 | 39:09:01:01 | 07:02:01:01 | 15:02:01:02 | 05:01:24:01 | 13:01:01:05 | 1.59 |
| 11 | 02:03:01:01 | 40:01:02:01 | 03:04:01:02 | 15:02:01:02 | 05:02:01:01 | 02:01:02:01 | 0.79 |
| 12 | 02:03:01:01 | 40:01:02:01 | 07:02:01:01 | 16:02:01:01 | 05:01:24:01 | 13:01:01:02 | 0.79 |
| 13 | 02:03:01:01 | 46:01:01:01 | 01:02:01:01 | 03:01:01:01 | 05:02:01:01 | 13:01:01:05 | 0.79 |
| 14 | 02:03:01:01 | 46:01:01:01 | 01:02:01:01 | 14:54:01:01 | 03:01:01:07 | 01:01:01:04 | 0.79 |
| 15 | 02:03:01:01 | 51:01:01:01 | 14:02:01:01 | 09:01:02:01 | 03:03:02:02 | 02:01:02:03 | 0.79 |
| 16 | 02:03:01:01 | 51:01:02:01 | 14:02:01:01 | 14:04:01:01 | 05:03:01:01 | 13:01:01:05 | 0.79 |
| 17 | 02:03:01:01 | 55:02:01:03 | 07:02:01:01 | 15:02:01:02 | 04:01:01:03 | 05:01:01:01 | 0.79 |
| 18 | 02:03:01:01 | 56:04:01:01 | 03:02:02:01 | 15:02:01:02 | 05:01:24:01 | 13:01:01:05 | 0.79 |
| 19 | 02:06:01:01 | 07:06:01:01 | 07:02:01:01 | 03:01:01:01 | 05:01:24:01 | 04:01:01:01 | 0.79 |
| 20 | 02:07:01:01 | 15:01:01:01 | 04:01:01:01 | 09:01:02:01 | 03:02:01:01 | 03:01:01:01 | 0.79 |
| 21 | 02:07:01:01 | 15:25:01:01 | 04:03:01:01 | 16:02:01:06 | 05:02:01:01 | 31:01:01:01 | 0.79 |
| 22 | 02:07:01:01 | 18:01:01:02 | 07:04:01:01 | 15:02:01:02 | 05:01:24:01 | 14:01:01:01 | 0.79 |
| 23 | 02:07:01:01 | 35:05:01:01 | 04:01:01:01 | 14:54:01:01 | 03:01:01:07 | 05:01:01:01 | 0.79 |
| 24 | 02:07:01:01 | 40:06:01:01 | 07:02:01:01 | 09:01:02:01 | 05:03:01:04 | 05:01:01:04 | 0.79 |
| 25 | 02:07:01:01 | 46:01:01:01 | 01:02:01:01 | 09:01:02:01 | 03:03:02:02 | 05:01:01:01 | 1.59 |
| 26 | 02:07:01:01 | 46:01:01:01 | 01:02:01:01 | 09:01:02:01 | 03:03:02:02 | 13:01:01:05 | 0.79 |
| 27 | 02:07:01:01 | 46:01:01:01 | 01:02:01:01 | 12:02:01:01 | 05:02:01:01 | 13:01:01:02 | 0.79 |
| 28 | 02:07:01:01 | 46:01:01:01 | 15:02:01:01 | 12:02:01:01 | 05:02:01:01 | 13:01:01:05 | 1.59 |
| 29 | 02:07:01:01 | 51:01:01:01 | 16:02:01:01 | 15:01:01:01 | 06:01:01:01 | 05:01:01:01 | 0.79 |
| 30 | 02:07:01:01 | 51:01:02:01 | 14:02:01:01 | 08:03:02:01 | 05:03:01:01 | 02:02:01:01 | 0.79 |
| 31 | 02:11:01:01 | 40:06:04:01 | 01:02:01:01 | 10:01:01:01 | 05:01:01:05 | 09:01:01:01 | 0.79 |
| 32 | 02:131:01:01 | 13:02:01:01 | 06:02:01:01 | 12:02:01:01 | 02:02:01:01 | 17:01:01:01 | 0.79 |
| 33 | 11:01:01:01 | 07:06:01:01 | 03:04:01:02 | 15:02:01:02 | 05:01:24:01 | 13:01:01:05 | 1.59 |
| 34 | 11:01:01:01 | 07:06:01:01 | 07:02:01:01 | 15:01:01:01 | 05:01:24:01 | 14:01:01:01 | 0.79 |
| 35 | 11:01:01:01 | 13:01:01:01 | 03:04:01:02 | 12:02:01:01 | 03:02:01:01 | 05:01:01:01 | 0.79 |
| 36 | 11:01:01:01 | 13:01:01:01 | 03:04:01:02 | 14:05:01:01 | 06:01:01:01 | 04:02:01:02 | 0.79 |
| 37 | 11:01:01:01 | 15:02:01:01 | 08:01:01:01 | 12:02:01:01 | 05:02:01:01 | 04:02:01:02 | 0.79 |
| 38 | 11:01:01:01 | 15:02:01:01 | 08:01:01:01 | 12:02:01:01 | 05:02:01:01 | 13:01:01:02 | 1.59 |
| 39 | 11:01:01:01 | 18:01:01:02 | 07:04:01:01 | 15:02:01:02 | 05:01:24:01 | 13:01:01:02 | 1.59 |
| 40 | 11:01:01:01 | 18:01:01:02 | 07:04:01:01 | 16:02:01:06 | 05:02:01:01 | 13:01:01:05 | 0.79 |
| 41 | 11:01:01:01 | 27:06:01:01 | 03:04:01:02 | 13:01:01:01 | 06:03:01:01 | 13:01:01:05 | 0.79 |
| 42 | 11:01:01:01 | 27:06:01:01 | 07:02:01:01 | 12:02:01:01 | 03:01:01:07 | 03:01:01:01 | 0.79 |
| 43 | 11:01:01:01 | 35:03:01:01 | 12:03:01:01 | 15:02:02:01 | 06:01:01:01 | 14:01:01:01 | 0.79 |
| 44 | 11:01:01:01 | 39:09:01:01 | 07:02:01:01 | 09:01:02:01 | 04:01:01:03 | 05:01:01:01 | 0.79 |
| 45 | 11:01:01:01 | 40:01:02:01 | 07:02:01:01 | 12:02:01:01 | 03:02:01:01 | 02:01:02:01 | 0.79 |
| 46 | 11:01:01:01 | 40:01:02:01 | 07:02:01:01 | 15:02:01:02 | 05:01:24:01 | 05:01:01:01 | 0.79 |
| 47 | 11:01:01:01 | 40:02:01:01 | 01:02:01:01 | 14:54:01:01 | 05:02:01:01 | 02:02:01:01 | 0.79 |
| 48 | 11:01:01:01 | 40:06:01:01 | 08:01:01:01 | 14:54:01:01 | 03:03:02:02 | 02:01:02:01 | 0.79 |
| 49 | 11:01:01:01 | 40:06:01:02 | 15:02:01:58 | 14:04:01:01 | 05:03:01:01 | 02:02:01:01 | 0.79 |
| 50 | 11:01:01:01 | 46:01:01:01 | 01:02:01:01 | 09:01:02:01 | 03:03:02:02 | 13:01:01:02 | 1.59 |
| 51 | 11:01:01:01 | 46:01:01:01 | 01:02:01:01 | 14:04:01:01 | 06:01:01:01 | 02:02:01:01 | 1.59 |
| 52 | 11:01:01:01 | 46:01:01:01 | 01:02:01:01 | 15:02:01:02 | 05:02:01:01 | 05:01:01:01 | 1.59 |
| 53 | 11:01:01:01 | 46:01:01:01 | 07:02:01:01 | 14:54:01:01 | 05:02:01:01 | 135:01:01:01 | 0.79 |
| 54 | 11:01:01:01 | 51:01:01:01 | 14:02:01:01 | 12:02:01:01 | 03:01:01:07 | 13:01:01:02 | 0.79 |
| 55 | 11:01:01:01 | 51:01:01:01 | 14:02:01:01 | 15:02:01:02 | 05:01:24:01 | 13:01:01:02 | 0.79 |
| 56 | 11:01:01:01 | 51:02:01:01 | 15:02:01:01 | 16:02:01:01 | 05:02:01:01 | 03:01:01:01 | 0.79 |
| 57 | 11:01:01:01 | 54:01:01:01 | 01:02:01:01 | 11:01:01:01 | 03:01:01:07 | 05:01:01:01 | 0.79 |
| 58 | 11:01:01:01 | 54:01:01:01 | 03:04:01:02 | 08:03:02:01 | 06:01:01:01 | 05:01:01:01 | 0.79 |
| 59 | 11:01:01:01 | 55:02:01:02 | 01:02:01:01 | 15:01:01:01 | 04:01:01:03 | 05:01:01:01 | 0.79 |
| 60 | 11:01:01:07 | 48:01:01:01 | 14:02:01:01 | 14:04:01:01 | 05:03:01:01 | 04:01:01:01 | 0.79 |
| 61 | 11:01:01:07 | 51:01:02:01 | 08:01:01:01 | 14:54:01:01 | 05:02:01:01 | 02:02:01:01 | 0.79 |
| 62 | 11:02:01:01 | 13:01:01:01 | 14:02:01:01 | 04:05:01:01 | 03:01:01:12 | 05:01:01:01 | 0.79 |
| 63 | 11:02:01:01 | 15:35:01:01 | 01:02:01:01 | 15:02:01:04 | 05:02:01:01 | 14:01:01:01 | 0.79 |
| 64 | 11:02:01:01 | 39:09:01:01 | 08:01:01:01 | 15:02:01:02 | 05:01:24:01 | 04:01:01:01 | 0.79 |
| 65 | 11:02:01:01 | 46:01:01:01 | 01:02:01:01 | 03:01:01:01 | 03:03:02:02 | 04:01:01:01 | 0.79 |
| 66 | 24:02:01:01 | 07:05:01:01 | 07:04:01:01 | 15:02:01:02 | 05:01:01:02 | 104:01:01:03 | 0.79 |
| 67 | 24:02:01:01 | 13:01:01:01 | 03:04:01:02 | 16:02:01:01 | 05:02:01:01 | 13:01:01:05 | 0.79 |
| 68 | 24:02:01:01 | 15:02:01:01 | 08:01:01:01 | 12:02:01:01 | 03:01:01:02 | 05:01:01:01 | 0.79 |
| 69 | 24:02:01:01 | 27:05:02:01 | 02:02:02:01 | 09:01:02:01 | 03:02:01:01 | 05:01:01:01 | 0.79 |
| 70 | 24:02:01:01 | 38:02:01:01 | 03:02:02:01 | 03:01:01:01 | 03:02:01:01 | 02:02:01:01 | 0.79 |
| 71 | 24:02:01:01 | 39:09:01:01 | 07:02:01:01 | 15:02:01:02 | 03:02:01:01 | 13:01:01:02 | 0.79 |
| 72 | 24:02:01:01 | 40:01:02:01 | 03:04:01:02 | 03:01:01:01 | 06:02:01:01 | 04:01:01:01 | 0.79 |
| 73 | 24:02:01:01 | 40:01:02:01 | 07:02:01:01 | 16:02:01:01 | 05:02:01:01 | 05:01:01:01 | 0.79 |
| 74 | 24:02:01:01 | 40:02:01:01 | 06:02:01:01 | 07:01:01:01 | 03:03:02:01 | 05:01:01:01 | 0.79 |
| 75 | 24:02:01:01 | 44:03:02:01 | 07:02:01:01 | 07:01:01:01 | 05:02:01:01 | 05:01:01:01 | 0.79 |
| 76 | 24:02:01:01 | 44:03:02:01 | 07:06:01:01 | 14:04:01:01 | 04:01:01:03 | 31:01:01:01 | 0.79 |
| 77 | 24:02:01:01 | 46:01:01:01 | 07:02:01:01 | 15:01:01:01 | 06:01:01:01 | 05:01:01:01 | 1.59 |
| 78 | 24:02:01:01 | 51:01:01:01 | 03:04:01:02 | 12:02:01:01 | 04:01:01:03 | 14:01:01:01 | 0.79 |
| 79 | 24:02:01:01 | 51:01:01:01 | 14:02:01:01 | 15:01:01:01 | 05:03:01:01 | 05:01:01:01 | 0.79 |
| 80 | 24:02:01:01 | 55:02:01:03 | 01:02:01:01 | 08:03:02:01 | 04:01:01:03 | 14:01:01:01 | 0.79 |
| 81 | 24:07:01:01 | 15:02:01:01 | 08:01:01:01 | 07:01:01:01 | 05:02:01:01 | 31:01:01:01 | 0.79 |
| 82 | 24:07:01:01 | 15:02:01:01 | 08:01:01:01 | 15:01:01:01 | 06:01:01:01 | 04:01:01:01 | 1.59 |
| 83 | 24:07:01:01 | 39:01:01:03 | 04:01:01:01 | 12:02:01:01 | 03:01:01:07 | 105:01:01:02 | 0.79 |
| 84 | 24:07:01:01 | 40:01:02:01 | 07:02:01:01 | 16:02:01:01 | 05:02:01:01 | 02:02:01:01 | 0.79 |
| 85 | 24:07:01:01 | 52:01:01:01 | 07:02:01:01 | 07:01:01:01 | 05:02:01:01 | 31:01:01:01 | 0.79 |
| 86 | 24:20:01:01 | 40:01:02:01 | 01:02:01:01 | 08:03:02:01 | 06:01:01:01 | 02:02:01:01 | 1.59 |
| 87 | 26:01:01:01 | 35:01:01:02 | 03:04:01:02 | 09:01:02:01 | 03:03:02:02 | 02:01:02:01 | 0.79 |
| 88 | 26:01:01:01 | 35:05:01:01 | 07:02:01:01 | 07:01:01:01 | 03:01:01:07 | 04:01:01:01 | 0.79 |
| 89 | 26:01:01:01 | 39:01:01:03 | 07:02:01:01 | 12:02:01:01 | 03:01:01:07 | 05:01:01:01 | 0.79 |
| 90 | 29:01:01:01 | 18:01:01:02 | 15:05:02:01 | 10:01:01:01 | 05:01:24:01 | 13:01:01:02 | 0.79 |
| 91 | 30:01:01:01 | 47:01:01:03 | 06:02:01:01 | 07:01:01:01 | 03:01:01:07 | 04:01:01:01 | 0.79 |
| 92 | 31:01:02:01 | 46:01:01:01 | 01:02:01:01 | 08:03:02:01 | 06:01:01:01 | 05:01:01:01 | 0.79 |
| 93 | 33:03:01:01 | 07:06:01:01 | 07:06:01:01 | 12:02:01:01 | 02:02:01:01 | 04:01:01:01 | 0.79 |
| 94 | 33:03:01:01 | 15:02:01:01 | 03:02:02:01 | 07:01:01:01 | 04:02:01:09 | 13:01:01:02 | 0.79 |
| 95 | 33:03:01:01 | 35:02:01:01 | 04:01:01:06 | 11:04:01:01 | 05:01:01:05 | 04:01:01:01 | 0.79 |
| 96 | 33:03:01:01 | 38:02:01:01 | 07:02:01:01 | 12:02:01:01 | 05:02:01:01 | 05:01:01:01 | 0.79 |
| 97 | 33:03:01:01 | 44:03:02:01 | 07:06:01:01 | 03:01:01:01 | 02:02:01:01 | 105:01:01:02 | 0.79 |
| 98 | 33:03:01:01 | 44:03:02:01 | 07:06:01:01 | 03:01:01:01 | 03:03:02:02 | 05:01:01:01 | 0.79 |
| 99 | 33:03:01:01 | 44:03:02:01 | 07:06:01:01 | 07:01:01:01 | 04:02:01:09 | 13:01:01:01 | 0.79 |
| 100 | 33:03:01:01 | 54:01:01:01 | 01:02:01:01 | 14:04:01:01 | 06:01:01:01 | 05:01:01:01 | 0.79 |
| 101 | 33:03:01:01 | 58:01:01:01 | 01:02:01:01 | 03:01:01:01 | 05:01:24:01 | 17:01:01:01 | 0.79 |
| 102 | 33:03:01:01 | 58:01:01:01 | 03:02:02:01 | 03:01:01:01 | 02:01:01:01 | 13:01:01:02 | 1.59 |
| 103 | 33:03:01:01 | 58:01:01:01 | 03:02:02:01 | 07:01:01:01 | 02:01:01:01 | 04:01:01:75 | 0.79 |
| 104 | 33:03:01:01 | 58:01:01:01 | 03:02:02:01 | 09:01:02:01 | 03:03:02:02 | 04:01:01:01 | 1.59 |
| 105 | 33:03:01:01 | 58:01:01:01 | 03:02:02:01 | 12:02:01:01 | 05:02:01:01 | 04:01:01:01 | 1.59 |
| 106 | 33:03:01:01 | 58:01:01:01 | 03:02:02:01 | 15:01:01:01 | 06:02:01:01 | 02:01:02:01 | 0.79 |
| 107 | 33:03:01:01 | 58:01:01:01 | 07:02:01:51 | 03:01:01:01 | 02:01:01:01 | 02:01:02:32 | 0.79 |
| 108 | 34:01:01:01 | 46:01:01:01 | 07:02:01:01 | 15:02:01:04 | 03:02:01:01 | 05:01:01:01 | 0.79 |
| 109 | 68:01:02:02 | 44:03:02:01 | 07:06:01:01 | 07:01:01:01 | 05:01:24:01 | 04:02:01:02 | 0.79 |
| 110 | 68:01:02:10 | 57:01:01:01 | 07:02:01:01 | 14:04:01:01 | 03:03:02:01 | 04:02:01:02 | 0.79 |
| 111 | 74:02:01:02 | 27:06:01:01 | 08:01:01:01 | 12:02:01:01 | 03:01:01:07 | 105:01:01:02 | 0.79 |
